# Public Money and Research

**Published:** 1919-02-01

**Authors:** 


### PUBLIC MONEY AND RESEARCH.

All the authorities tell us that "research" must be pursued. They tell us, too, that it must be encouraged, and this, in plain blunt terms, means not only an occupation, but also a salary. We agree; but the question is, Where is the salary to come from? The general reply, we suppose, would be—the public purse. Unfortunately just now this purse is not a very full one, and there are many applicants for its scanty contents. This, however, is not the main difficulty. Public money can be found when an imperative necessity is shown, as the financial history of the war prominently proclaims. The real difficulty in the application of public money for purposes of research is the definition of the object and the checking of the result. Rumour has it that even in Government enterprises directed to severely material ends extravagance occasionally creeps in, but even here there is an account to be rendered and balanced. The activity, at least in some degree, comes out in figures, and may be judged by the ends accomplished. The worth of a Cabinet Minister falls under the same kind of test, though custom prescribes that the judgment be exercised with charity. But "research" quite frankly and openly refuses such a criterion, and we are not the least inclined to quarrel with the decision. Still the fact is a hard one for a Minister charged to defend public expenditure before the critical economists of the House of Commons—that is, it is a hard one if he has spent money on research and has nothing to announce for his pains. In such circumstances there will always be a pressure to produce "results," and the history of science shows more than one disastrous outcome of this vice. Research, we are told by all the authorities, must be free in regard both to time and outcome, and even when the issue is negative the process has its justification. As a general doctrine this may be all right, but there must be a limit to it, for no one can propose an unending public subsidy to a person or a process that never produces any return. A discoverer who does not discover cannot continue indefinitely as a public charge. Yet payment by results is dismissed with scorn by some of the most energetic advocates of the endowment of research.

We are afraid that the difficulties of the position have not been made less by some recent communications to the Press. One gentleman has told us that the true researcher has to spend 95 per cent. of his time in thinking, and only 5 per cent. of it in practical work. If the public have to find the money for such an enterprise it is to be feared that rude voices will ask for a guarantee that the thinking is actually performed; and this brings us once more face to face with the objection to payment by results. In practice, and so far as public money is concerned, we do not think that this test can be avoided, whatever academic protests may be made in opposition. And in the majority of instances it will serve. There will remain the great enterprises and those who undertake them because they can do no other. It may be said of the discoverer and inventor, as of the poet, that he is born, not made. A measure of success is open to industry and talent, but genius frames its own flight, and even a Government subsidy will neither inspire nor repress it.

